# Unveiling the mystery: Investigating the debate surrounding mitochondrial DNA copy number and Sjögren syndrome using Mendelian randomization analysis

**DOI:** 10.1097/MD.0000000000040908

**Published:** 2024-12-13

**Authors:** Jie Zhou, Yixin Xu, Haitao Wang, Chao Chen, Kun Wang

**Affiliations:** a The Wujin Hospital Affiliated with Jiangsu University, Changzhou, China; b The Wujin Clinical College of Xuzhou Medical University, Changzhou, China; c The Third Affiliated Hospital of Soochow University, Changzhou, China.

**Keywords:** autoimmune disease, causal relationship, genetically predicted, GWAS, single nucleotide polymorphism

## Abstract

Numerous studies have investigated the relationship between mitochondrial DNA (mtDNA) copy number and Sjögren syndrome (SS). However, the conclusions remain inconclusive, with conflicting findings. The genome-wide association study summary statistics for mtDNA copy number were obtained from 2 sources: a cohort of 465,809 White individuals from the Cohorts for Heart and Aging Research in Genomic Epidemiology consortium and the UK Biobank, and a dataset of 395,718 UK Biobank participants. Additionally, we obtained 2 sets of genome-wide association study summary statistics for SS through datasets from FinnGen and the UK Biobank, involving a total of 809,836 participants. Furthermore, we conducted a two-sample bidirectional Mendelian randomization analysis, primarily utilizing the inverse variance weighted method, complemented by 4 other validation methods, to explore the association between mtDNA copy number and SS. Following our comprehensive investigation, no discernible causal relationship was identified between mtDNA copy number and SS in either the training or validation cohorts (inverse variance weighted, *P* *>* .05). Similarly, the reverse Mendelian randomization analysis yielded negative results (inverse variance weighted, *P* *>* .05). Furthermore, all analyses indicated an absence of horizontal pleiotropy or heterogeneity. Our analysis revealed no causal relationship between mtDNA copy number and SS.

## 1. Introduction

Sjögren syndrome (SS) is a systemic autoimmune disease characterized by inflammation of the exocrine glands, primarily affecting the salivary and lacrimal glands.^[1]^ According to statistics, the incidence of SS ranges from 3 to 11 cases per 100,000 individuals,^[2,3]^ with a prevalence ranging from 0.01% to 0.72%.^[4,5]^ Additionally, SS can be a serious condition associated with a higher mortality rate. Traditional causes of elevated mortality in SS patients include B cell lymphoma, severe organ-specific complications (mainly interstitial lung disease, renal failure, and severe cryoglobulinemic vasculitis), as well as infections and cardiovascular diseases.^[6]^

The current theory regarding the pathogenesis of SS is known as “autoimmune epithelitis,” suggesting that epithelial tissues serve as the primary focus of inflammation in SS.^[7]^ The involvement of organ exocrine pathology and functional impairment, particularly in the parenchyma, is associated with lymphocytic infiltration surrounding or infiltrating organs in close proximity to epithelial cells.^[1]^ Research indicates that salivary gland epithelial cells possess the capability to detect innate immune signals through Toll-like receptors, resulting in the upregulation of immune-related molecules such as Human Leukocyte Antigen class I molecules, FAS receptor, FAS ligand, and pro-inflammatory cytokines within the glandular epithelium.^[8,9]^ Furthermore, apoptosis of glandular epithelial cells has been recognized as a potential pathogenic mechanism linked to secretory gland dysfunction.^[10]^

Mitochondria play a crucial role in the fundamental functions of eukaryotic cells. An increasing body of evidence suggests that many signaling pathways are closely intertwined with cellular metabolism. Metabolism not only provides fuel for active cells but also guides decisions regarding cellular fate.^[11]^ Therefore, mitochondria are extensively involved in energy production, reactive oxygen species regulation, autophagy, aging, and participation in cellular signaling pathways.^[12]^ Metabolism not only maintains different immune cell phenotypes due to changes in cell signaling but also feedback and alter signals to drive immune cell phenotypes.^[11]^ Mitochondria serve as the central hub of metabolism, making them essential for maintaining and establishing immune cell phenotypes. In recent years, there has been a growing recognition of the critical role of mitochondrial signaling in determining macrophage polarization and function,^[13]^ responding to innate immune signal activation,^[14]^ and controlling adaptive immunity (such as T cell activation, CD4 T cell differentiation, and CD8 memory T cell formation).^[11]^

Moreover, each mitochondrion has multiple copies of mitochondrial DNA (mtDNA). The regulation of mtDNA copy number is tightly controlled to ensure that mitochondria can generate adequate energy and intracellular signals to maintain normal cellular function.^[15]^ In recent years, mitochondrial dysfunction has been found to be associated with autoimmune diseases such as systemic lupus erythematosus,^[16]^ multiple sclerosis,^[17]^ rheumatoid arthritis,^[18]^ and type 1 diabetes.^[19]^ Furthermore, changes in mitochondrial morphology have been observed in the salivary gland epithelial cells of SS patients. This finding reinforces the correlation between the 2.^[20]^ However, a significant contradiction persists regarding the relationship between SS and mtDNA copy number. For instance, Benedittis et al conducted an analysis of mtDNA copy number in the peripheral blood of 74 SS patients and 61 healthy controls using qPCR. Their findings revealed a noteworthy decrease in mtDNA copy number among SS patients (*P* *=* 1.44 × 10^−12^).^[21]^ What adds to the puzzle is the discovery by Zhao et al, who, after examining 89 female participants with SS and 98 gender-matched healthy controls, observed an increase in mtDNA copy number in the peripheral blood of SS patients.^[22]^

The evidence supporting a causal relationship between mtDNA copy number and SS is currently limited to observational studies. To address the constraints of such studies, Mendelian randomization (MR) utilizing data from genome-wide association studies (GWAS) emerges as a promising approach to evaluate causality within a hypothetical exposure-outcome pathway.^[23]^ In essence, MR functions as nature’s randomized trial, leveraging the fortuitous allocation of genetic variants at conception to categorize individuals into distinct subgroups akin to a placebo and intervention group in a randomized controlled trial. This methodology enables the examination of potential causal links between risk factors (e.g., mtDNA copy number) and disease outcomes (e.g., SS) while ensuring that confounding variables are also randomized.^[24]^ In our study, we have compiled several recently published GWAS summary statistics concerning SS and mtDNA copy number. Through a two-sample MR analysis, our primary objective is to unveil the causal relationship between mtDNA copy number and SS. By doing so, we aim to shed light on the pathogenesis of SS and identify potential therapeutic targets.

## 2. Methods

### 2.1. Study design

In this study, all data were obtained from publicly available databases and have been approved by the relevant research institution’s review board. Therefore, ethical committee review is not required for this study.

In this research, we employed single nucleotide polymorphisms (SNPs) as instrumental variables (IVs) to conduct a thorough bidirectional MR analysis,^[25]^ investigating the causal relationship between mtDNA copy number and SS. Furthermore, MR analysis must adhere to 3 key assumptions: (1) SNPs are associated with the exposure; (2) SNPs are independent of confounding factors in the exposure-outcome relationship; and (3) SNPs influence the outcome solely through the exposure.^[26]^

### 2.2. GWAS summary data sources

#### 2.2.1. mtDNA copy number

We conducted our analysis using GWAS summary statistics data on mtDNA copy number from 2 groups. The training group’s GWAS data on mtDNA copy number is derived from a robust cohort comprising 465,809 individuals of European descent, sourced from the esteemed Cohorts for Heart and Aging Research in Genomic Epidemiology and the UK Biobank. Longchamps et al provided detailed descriptions of this cohort in their 2022 publication.^[27]^ To enhance the credibility of our research findings, the validation group’s GWAS data for mtDNA copy number consists of 395,718 participants from the UK Biobank, each with diverse ancestral backgrounds primarily of European descent.^[28]^ The GWAS included adjustments for age, age squared, sex, chip type, 20 genetic principal components, and blood cell counts (white blood cell, platelet, and neutrophil counts), providing a more comprehensive genetic assessment of mtDNA copy number compared to prior studies.^[27,29]^ The information about each data source is provided in Table 1.

**Table 1:** Details of the genome-wide association studies and datasets used in our analyses.

Phenotypes	Cases/Controls	Consortium/Author	PubMed ID	Data download link
Mitochondrial DNA copy number	395,718	UK Biobank	35023831	https://www.ebi.ac.uk/gwas/
Mitochondrial DNA copy number in the replication analysis	465,809	CHARGE and UK Biobank	34859289	https://www.ebi.ac.uk/gwas/
Sjögren syndrome	2735/399,355	FinnGen consortium	–	https://storage.googleapis.com/finngen-public-data-r10/summary_stats/finngen_R10_M13_SJOGREN.gz
Sjögren syndrome	407,746	UK Biobank	34017140	https://gwas.mrcieu.ac.uk/; ID: ebi-a-GCST90013929

#### 2.2.2. Sjögren syndrome

The summary data for one of the cohorts on SS was sourced from FinnGen Release 10 (DF10, Public release: December 18, 2023), consisting of a total of 402,090 samples (2735 cases; 399,355 controls; https://storage.googleapis.com/finngen-public-data-r10/summary_stats/R10_manifest.tsv). To validate the accuracy of our findings, we have integrated an additional set of GWAS data on SS. This dataset originates from the UK Biobank, encompassing information on up to 407,746 individuals. The summary data can be accessed at https://gwas.mrcieu.ac.uk.^[30]^ The information about each data source is provided in Table 1.

### 2.3. IVs selection and data harmonization

In our study, we conducted rigorous screening of IVs to ensure high standards. Firstly, we only included SNPs with genome-wide significance (*P* *<* 5 × 10^−8^) as our study subjects. Secondly, in the MR analysis, we excluded palindromic and ambiguous SNPs as IVs.^[31]^ Subsequently, we grouped SNPs based on linkage disequilibrium with a window size of 10,000 kb and r^2^ < 0.001. Additionally, we calculated the F-statistic to assess the variance explained by each exposure SNP, using the formula [(N − K − 1)/K]/[R^2^/ (1 − R^2^)], where K represents the number of genetic instruments and N is the sample size.^[32]^ To ensure the reliability and stability of our results, we excluded weak IVs with an F value <10. Finally, we used the online tool PhenoScanner to assess all established phenotypes associated with the genetic instruments considered in our study, manually excluding SNPs associated with confounding factors (http://www.phenoscanner.medschl.cam.ac.uk/).^[33]^

### 2.4. Statistical analysis

For our meticulous and thorough analysis, we conducted MR analysis using R software (version 4.2.0, http://www.r-project.org) in conjunction with the “Two-Sample MR” package (version 0.5.6).^[34]^

### 2.5. Primary analysis

To explore the causal relationship between mtDNA copy number and SS, we conducted a two-sample MR analysis. Initially, we utilized mtDNA copy number data from the training cohort as the exposure and 2 distinct SS datasets as outcomes for the MR analysis. Subsequently, we validated these results using mtDNA copy number data from the validation cohort.

Furthermore, in the current MR analysis, we employed a variety of techniques, including inverse variance weighted (IVW), MR-Egger, weighted median, weighted mode, and simple mode. Additionally, a significance level of *P* *<* .05 indicates a positive result. More specifically, IVW, as a key research method, combines a meta-analysis strategy with the Wald estimate for each SNP. In the absence of horizontal pleiotropy, the results from IVW are unbiased.^[35]^ Based on the instrument strength independent of direct effect assumption, MR-Egger regression assesses pleiotropy through the intercept term. If the intercept term is zero, the results from MR-Egger regression align with IVW, indicating the absence of horizontal pleiotropy.^[36]^ When up to 50% of IVs are invalid, the weighted median technique accurately evaluates causal relationships.^[37]^ Research has shown that in cases where the instrument strength independent of direct effect assumption is violated, the weighted mode estimate has greater power to identify causal effects, lower bias, and lower type I error rates compared to MR-Egger regression.^[37]^ Additionally, the simple mode method, despite its lower precision, reduces bias.^[37]^ These techniques are crucial for ensuring the reliability of research findings.

### 2.6. Reverse MR analysis

To investigate the potential reverse causal relationship between mtDNA copy number and SS, this study conducted a reverse MR analysis, considering SS as the exposure and mtDNA copy number as the outcome. The aforementioned techniques were employed for the analysis, and the robustness of the findings was further confirmed by utilizing data from a validation cohort.

### 2.7. Sensitivity analysis

Due to differences in experimental conditions, study populations, and SNPs, there may be heterogeneity in two-sample MR analysis, potentially leading to bias in estimating causal effects.

Therefore, this study conducted heterogeneity tests using the IVW and MR-Egger methods. Cochrane Q statistic was used to assess the heterogeneity of genetic instruments, with a *P-value* *>* .05 indicating no significant heterogeneity.^[38]^ Additionally, a fundamental assumption in MR analysis is that the IV affects the outcome only through the exposure, necessitating investigation into potential horizontal pleiotropy between exposure and outcome.^[39]^ This study employed the MR-Egger intercept method to assess the presence of pleiotropy. A *P-value* *>* .05 suggests minimal or negligible potential for pleiotropy in causal analysis, allowing for its exclusion. Finally, outliers in the IVW analysis were identified and adjusted using the MR-PRESSO test,^[40]^ while the leave-one-out analysis was used to determine the genetic causal effects of individual SNPs on the exposure-outcome relationship.^[41]^

## 3. Results

### 3.1. Association of genetically predicted mtDNA copy number with SS in training cohort

In the training cohort, after conducting LD clumping, exploring proxy SNPs, mining the Phenoscanner database, and harmonizing the data, we selected eligible SNPs as IVs to meet 3 key assumptions. Specifically, a total of 70 SNPs associated with mtDNA copy number were obtained as IVs for further analysis (Table S1, Supplemental Digital Content, http://links.lww.com/MD/O169).

To explore the genetically predicted causal relationship between mtDNA copy number and SS, we leveraged the mtDNA copy number dataset from the training cohort as the exposure and utilized datasets from 2 SS cohorts as the outcomes for MR analysis (Table S2, Supplemental Digital Content, http://links.lww.com/MD/O169).

**Figure 1.**
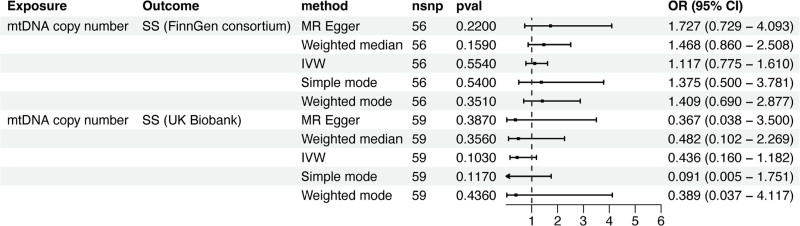
The MR analysis between mtDNA copy number and SS in the training cohort. Note: MR, Mendelian randomization; mtDNA, mitochondrial DNA; SS, Sjögren syndrome; nSNPs, number of single nucleotide polymorphisms; OR, odds ratio; or_lci95, 95% lower confidence interval; or_uci95, 95% upper confidence interval; IVW, inverse variance weighted.

In the FinnGen GWAS data for SS, characterized by the phenotype of Sicca syndrome, a comprehensive MR analysis failed to reveal any significant evidence of a genetically predicted causal association between mtDNA copy number and SS. The results were as follows: IVW method: odds ratio (OR) 1.117; 95% confidence interval (CI) 0.775–1.610; *P* *=* .554; MR Egger method: OR 1.727; 95% CI 0.729–4.093; *P* *=* .220; Weighted median method: OR 1.468; 95% CI 0.860–2.508; *P* *=* .159; Simple mode: OR 1.375; 95% CI 0.500–3.781; *P* *=* .540; Weighted mode: OR 1.409; 95% CI 0.690–2.877; *P* *=* .351 (Fig. 1).

Furthermore, a detailed analysis was conducted using a secondary dataset from the UK Biobank to explore the relationship between mtDNA copy number and SS. Unfortunately, our findings did not reveal any conclusive causal link between these variables: IVW method: OR 0.436; 95% CI 0.160–1.182; *P* *=* .103; MR Egger method: OR 0.367; 95% CI 0.038–3.500; *P* *=* .387; weighted median method: OR 0.482; 95% CI 0.102–2.269; *P* *=* .356; simple mode: OR 0.091; 95% CI 0.005–1.751; *P* *=* .117; and weighted mode: OR 0.389; 95% CI 0.037–4.117; *P* *=* .436 (Fig. 1).

### 3.2. Reverse MR analysis in the training cohort

To explore the potential for reverse causality, we performed a reverse MR analysis, where we considered SS as the exposure and mtDNA copy number in the training cohort as the outcome.

**Figure 2.**
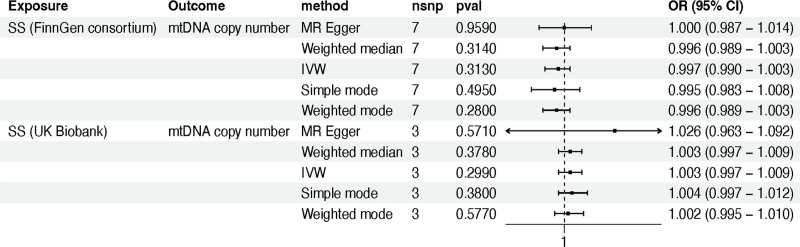
The reverse MR analysis between mtDNA copy number and SS in the training cohort. Note: MR, Mendelian randomization; mtDNA, mitochondrial DNA; SS, Sjögren syndrome; nSNPs, number of single nucleotide polymorphisms; OR, odds ratio; or_lci95, 95% lower confidence interval; or_uci95, 95% upper confidence interval; IVW, inverse variance weighted.

In the FinnGen dataset, our reverse MR analysis did not show a causal relationship between SS and mtDNA copy number (IVW method: OR 0.997; 95% CI 0.990–1.003; *P* *=* .313) (Table S3, Supplemental Digital Content, http://links.lww.com/MD/O169; Fig. 2). Similarly, when using a different dataset for SS, we also did not find evidence of causality between the 2 variables (IVW method: OR 1.003; 95% CI 0.997–1.009; *P* *=* .299). These consistent significant associations were further supported by MR Egger, weighted median, simple mode, and weighted mode methods (*P* *>* .05) (Table S3, Supplemental Digital Content, http://links.lww.com/MD/O169; Fig. 2).

### 3.3. Conducting bidirectional MR analysis in the validation cohort

We conducted a validation analysis using GWAS summary statistics for mtDNA copy number from 395,718 UK Biobank participants, mostly of European descent. Following LD clumping, proxy SNP exploration, Phenoscanner database searches, and data harmonization, 68 SNPs linked to mtDNA copy number were chosen for additional analysis in the validation cohort (Table S4, Supplemental Digital Content, http://links.lww.com/MD/O169).

**Figure 3.**
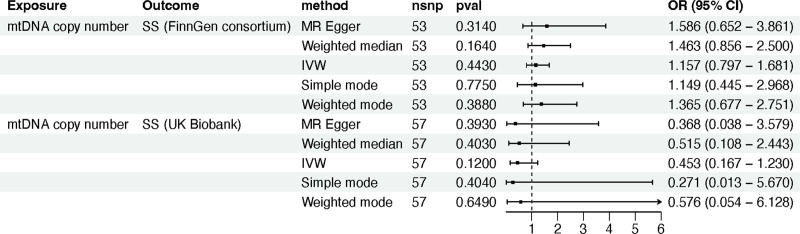
The MR analysis between mtDNA copy number and Sjögren syndrome in the validation cohort. Note: MR, Mendelian randomization; mtDNA, mitochondrial DNA; SS, Sjögren syndrome; nSNPs, number of single nucleotide polymorphisms; OR, odds ratio; or_lci95, 95% lower confidence interval; or_uci95, 95% upper confidence interval; IVW, inverse variance weighted.

In a parallel fashion, we conducted MR analysis utilizing the mtDNA copy number dataset from the validation cohort as the exposure and the SS dataset from the FinnGen cohort as the outcome. The findings mirrored those of the training cohort, demonstrating an absence of any causal relationship between mtDNA copy number and SS (IVW method: OR 1.157; 95% CI 0.797–1.681; *P* *=* .443) (Table S5, Supplemental Digital Content, http://links.lww.com/MD/O169; Fig. 3). This consensus was echoed in the MR analysis of an additional SS dataset (IVW method: OR 0.453; 95% CI 0.167–1.230; *P* *=* .120) (Table S5, Supplemental Digital Content, http://links.lww.com/MD/O169; Fig. 3).

**Figure 4.**
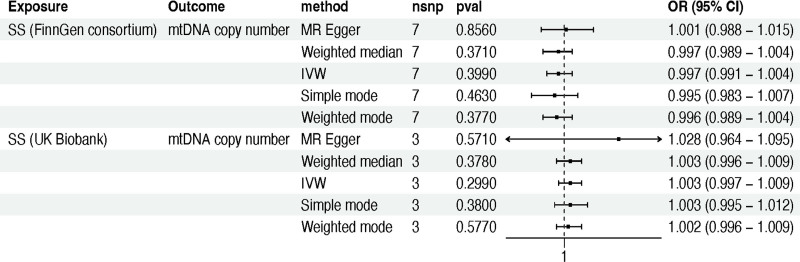
The reverse MR analysis between mtDNA copy number and Sjögren syndrome in the validation cohort. Note: MR, Mendelian randomization; mtDNA, mitochondrial DNA; SS, Sjögren syndrome; nSNPs, number of single nucleotide polymorphisms; OR, odds ratio; or_lci95, 95% lower confidence interval; or_uci95, 95% upper confidence interval; IVW, inverse variance weighted.

Subsequent reverse MR analysis, with SS as the exposure variable and mtDNA copy number from the validation set as the outcome variable, failed to yield any statistically significant results in both datasets (IVW_FinnGen_ method: OR 0.997; 95% CI 0.991–1.004; *P* *=* .399; IVW_UK Biobank_ method: OR 1.003; 95% CI 0.997–1.009; *P* *=* .299) (Table S6, Supplemental Digital Content, http://links.lww.com/MD/O169; Fig. 4).

These coherent and statistically significant associations were further substantiated by the application of MR Egger, weighted median, simple mode, and weighted mode methods (*P* > .05) (Tables S5 and S6, Supplemental Digital Content, http://links.lww.com/MD/O169).

### 3.4. Sensitivity analysis

In the analyses mentioned above, we utilized the online tool PhenoScanner to evaluate each SNP, and the results revealed no association between these SNPs and the investigated outcomes. During outlier detection, we found no outliers in any of the forward MR analyses. In the reverse MR analysis, no outliers were identified when analyzing SS data from FinnGen as the exposure. However, due to the limited number of IVs, outlier detection was not feasible when using SS data from the UK Biobank as the exposure. Moreover, across all analyses encompassing the training cohort, validation cohort, and all reverse MR analyses, no indications of heterogeneity or horizontal pleiotropy were observed (Table S7A and B, Supplemental Digital Content, http://links.lww.com/MD/O169). Finally, we employed a “leave-one-out” approach for sensitivity analysis to investigate whether specific SNPs influenced the causal relationships. The results indicated that systematically excluding each SNP did not lead to significant changes in the model’s effect estimates or qualitative conclusions (Figs. S1–S4, Supplemental Digital Content, http://links.lww.com/MD/O170).

## 4. Discussion

SS, as an autoimmune disease characterized by inflammation of exocrine glands, involves organ pathology and functional impairment closely associated with lymphocytic infiltration surrounding or infiltrating epithelial cells.^[1]^ Mitochondria, as central hubs of metabolism, are crucial for maintaining and establishing immune cell phenotypes. The roles of mitochondria in activating innate immune signals and regulating adaptive immunity are being increasingly explored.^[11]^ Additionally, mitochondria play vital roles in regulating reactive oxygen species signaling, energy production, calcium homeostasis, and cell apoptosis.^[42]^ mtDNA copy number may respond to physiological or environmental stimuli along with increases in reactive oxygen species.^[43,44]^ Consequently, researchers have sought to explore the relationship between mtDNA copy number and SS. However, due to the impact of various confounding factors, studies in this field have yielded inconsistent and conflicting results.^[21,22]^ In this study, we have innovatively utilized existing GWAS data to introduce a novel approach. By employing MR analysis, we aim to establish a correlation between mtDNA copy number and SS. Through the application of two-sample bidirectional MR analysis, our objective is to delve into the causal relationship between these 2 factors and pinpoint potential therapeutic targets for SS.

In previous studies exploring the relationship between mtDNA copy number and SS, researchers consistently found variations in mtDNA copy number among SS patients. However, Benedittis et al revealed a significant decrease in mtDNA copy number in SS patients,^[21]^ while Zhao et al observed an increase in mtDNA copy number in the peripheral blood of SS patients.^[22]^ Controversies in scientific research often arise from the influence of confounding factors. Firstly, regarding the demographics and sample sizes, Benedittis et al primarily investigated European populations, whereas Zhao et al focused on East Asian populations, with both studies enrolling fewer than 100 SS patients.^[21,22]^ Secondly, while both investigations identified variations in mtDNA copy numbers between SS patients and healthy controls, the extent of these differences was modest, potentially contributing to the divergent findings.^[21,22]^ Moreover, both approaches employed real-time quantitative polymerase chain reaction for assessing mtDNA copy numbers. Nonetheless, they exhibited differences in DNA extraction techniques, primer selection, experimental protocols, and formulas for calculating copy numbers, all of which could affect the precision and reliability of the results.^[21,22]^ However, our study analyzed GWAS summary statistics for 2 groups of SS and 2 groups of mtDNA copy number. Through MR analysis, we did not find any causal relationship between mtDNA copy number and SS in both the training and validation cohorts. Similarly, reverse MR analysis yielded negative results in both the training and validation cohorts. Furthermore, tests for horizontal pleiotropy, heterogeneity analysis, and sensitivity analysis all met the 3 core assumptions of MR, further validating the reliability of our findings. These findings are inconsistent with previous research results.

The presence of numerous confounding factors often leads to inconsistent research results. The observed differences in mtDNA copy numbers may be attributed to the use of different methods for estimating mtDNA content and DNA extraction. Therefore, to understand the significance of mtDNA copy number changes in SS, standardized methods should be used to calculate mtDNA copy number. Additionally, factors such as regional differences, study populations, sample sizes, statistical variations, and technical limitations can all contribute to biases in research findings. Therefore, by using genetic variation as an IV, MR can help mitigate the impact of unmeasured confounders or biases. This approach can allow for inferences to be drawn from observational data regarding the impact of mtDNA copy number on SS, thereby enhancing the credibility of the conclusions.

Based on our understanding, our study represents the most comprehensive, largest-scale, and earliest MR analysis conducted on the genetic causal effect of mtDNA copy number on the risk of SS. Our MR study has several strengths. Firstly, we employed a two-sample MR design to mitigate the effects of common confounders and reverse causation often observed in traditional observational studies. Additionally, we carefully selected IVs related to the exposure, strictly adhering to the fundamental assumptions of MR analysis. Lastly, our study encompassed analyses from multiple datasets and validated the research findings using a validation cohort, with consistent results supporting our conclusions.

Our study has several limitations. Firstly, as our genetic analyses primarily focused on patients of European descent, the generalizability of our research findings to other ethnicities may be limited. Secondly, despite conducting various sensitivity analyses, we were unable to completely eliminate the potential impact of polymorphisms or heterogeneity.

## Author contributions

**Conceptualization:** Jie Zhou.

**Data curation:** Jie Zhou, Haitao Wang, Kun Wang, Chao Chen.

**Formal analysis:** Jie Zhou, Kun Wang.

**Funding acquisition:** Yixin Xu.

**Investigation:** Yixin Xu, Chao Chen.

**Methodology:** Jie Zhou, Yixin Xu, Kun Wang.

**Resources:** Chao Chen.

**Software:** Jie Zhou.

**Supervision:** Yixin Xu, Haitao Wang.

**Validation:** Yixin Xu, Haitao Wang.

**Writing – original draft:** Jie Zhou.

## Supplementary Material


