# Supplementary material for: Unveiling the mystery: Investigating the debate surrounding mitochondrial DNA copy number and Sjögren syndrome using Mendelian randomization analysis
Source: Medicine (Baltimore). 2024 Dec 13;103(50):e40908. doi: 10.1097/MD.0000000000040908 (PMC11651504; doi:10.1097/MD.0000000000040908)
Supplement: Supplementary file 2 [file medi-103-e40908-s002.pdf]

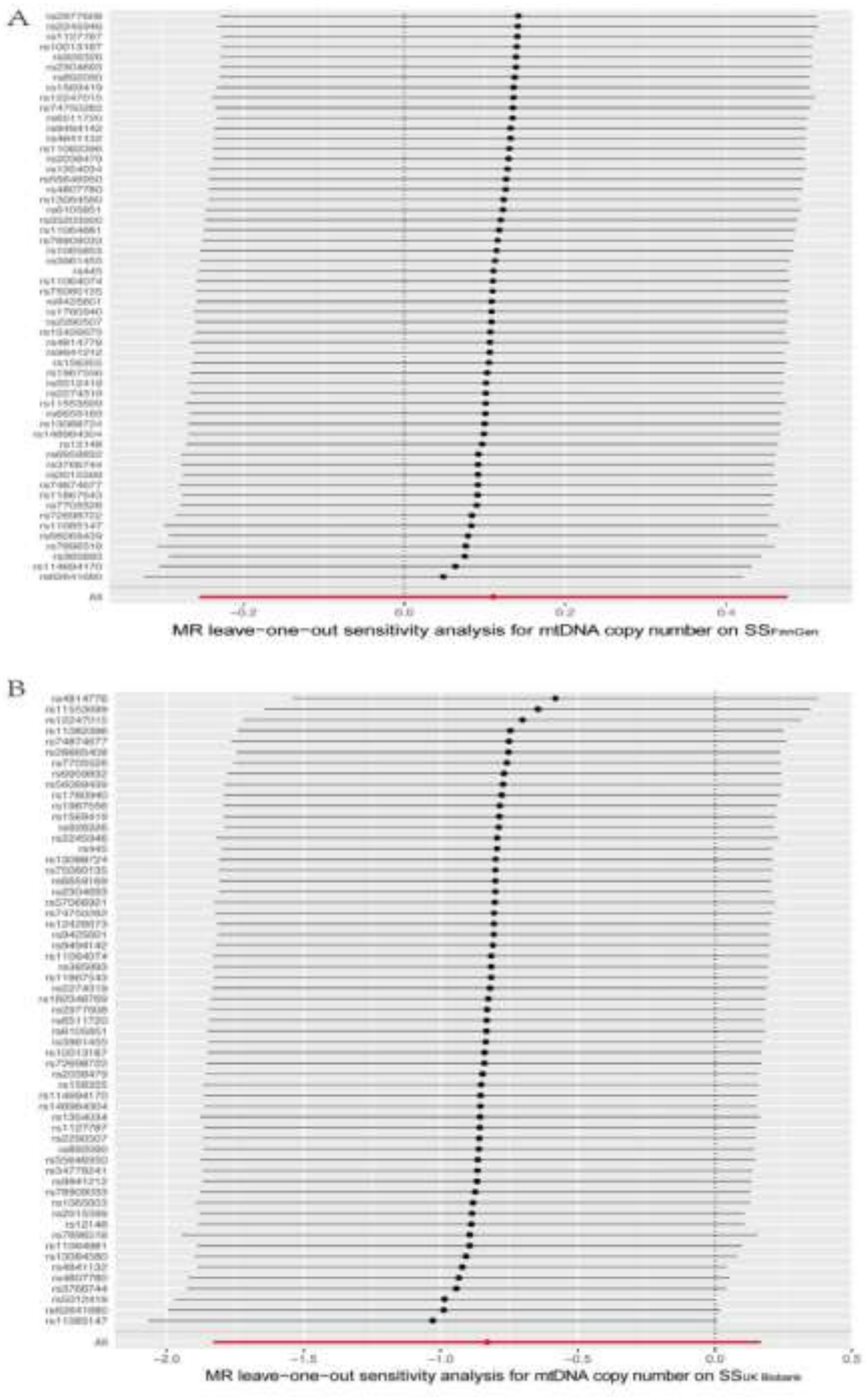

Supplementary Figure S1: Leave-one-out plot to visualize causal effect of mtDNA copy number on SS risk when leaving out one SNP in the training cohort. A, SS data from FinnGen; B, SS data from UK Biobank; mtDNA, mitochondrial DNA; SS, Sjogren's syndrome; MR, Mendelian randomization.

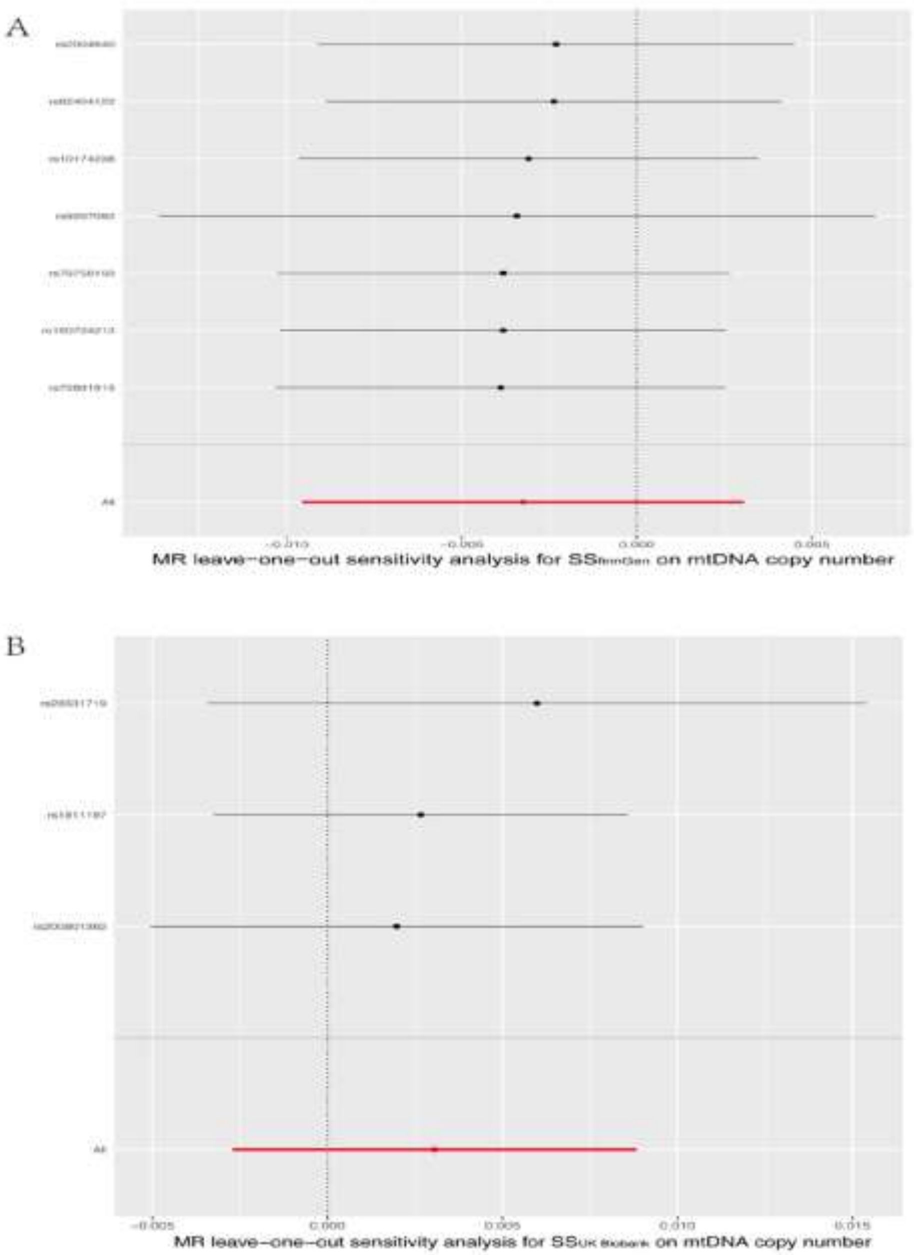

Supplementary Figure S2: Leave-one-out plot to visualize causal effect of SS risk on mtDNA copy number when leaving out one SNP in the training cohort. A, SS data from FinnGen; B, SS data from UK Biobank; mtDNA, mitochondrial DNA; SS, Sjogren's syndrome; MR, Mendelian randomization.

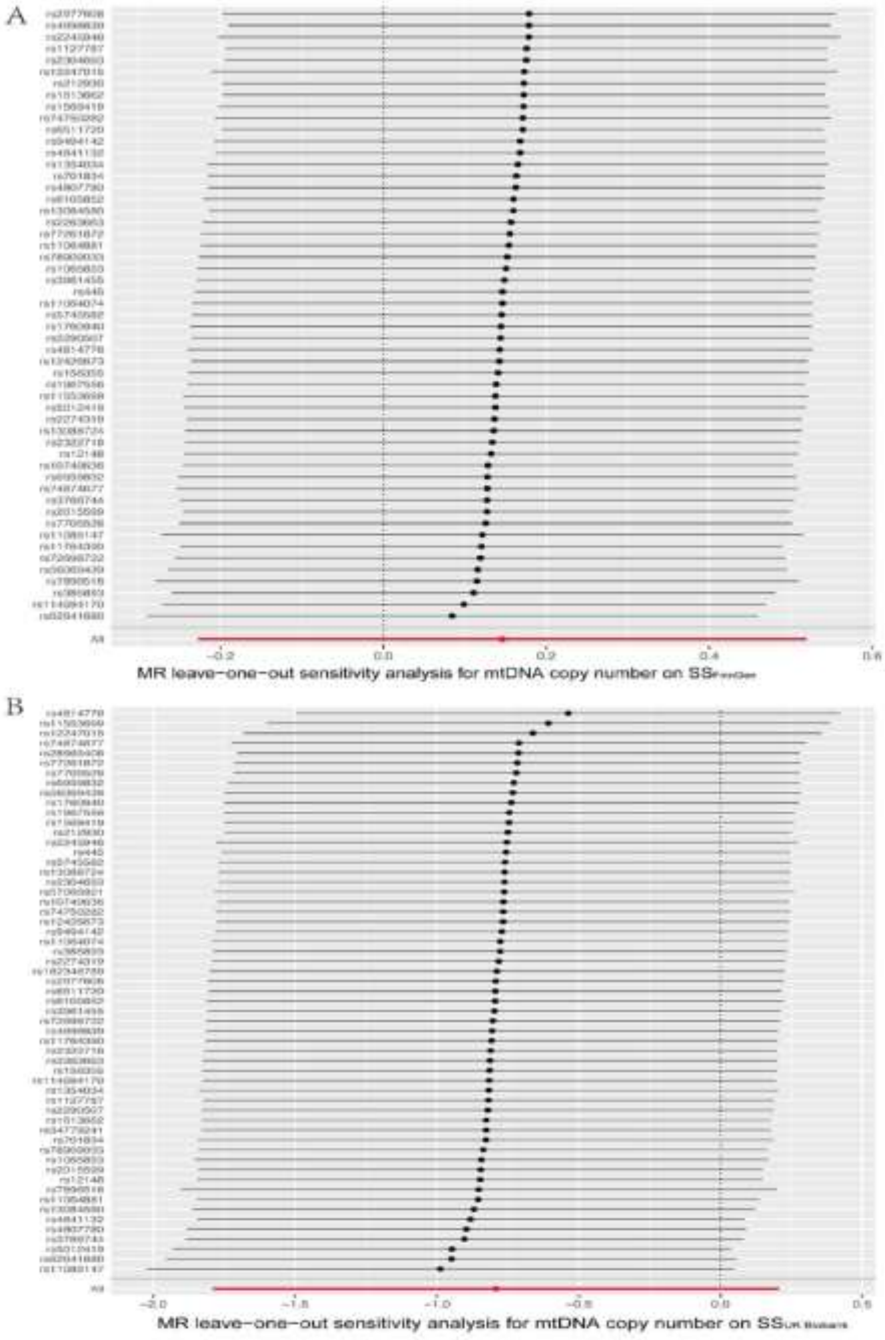

Supplementary Figure S3: Leave-one-out plot to visualize causal effect of mtDNA copy number on SS risk when leaving out one SNP in the validation cohort. A, SS data from FinnGen; B, SS data from UK Biobank; mtDNA, mitochondrial DNA; SS, Sjogren's syndrome; MR, Mendelian randomization.

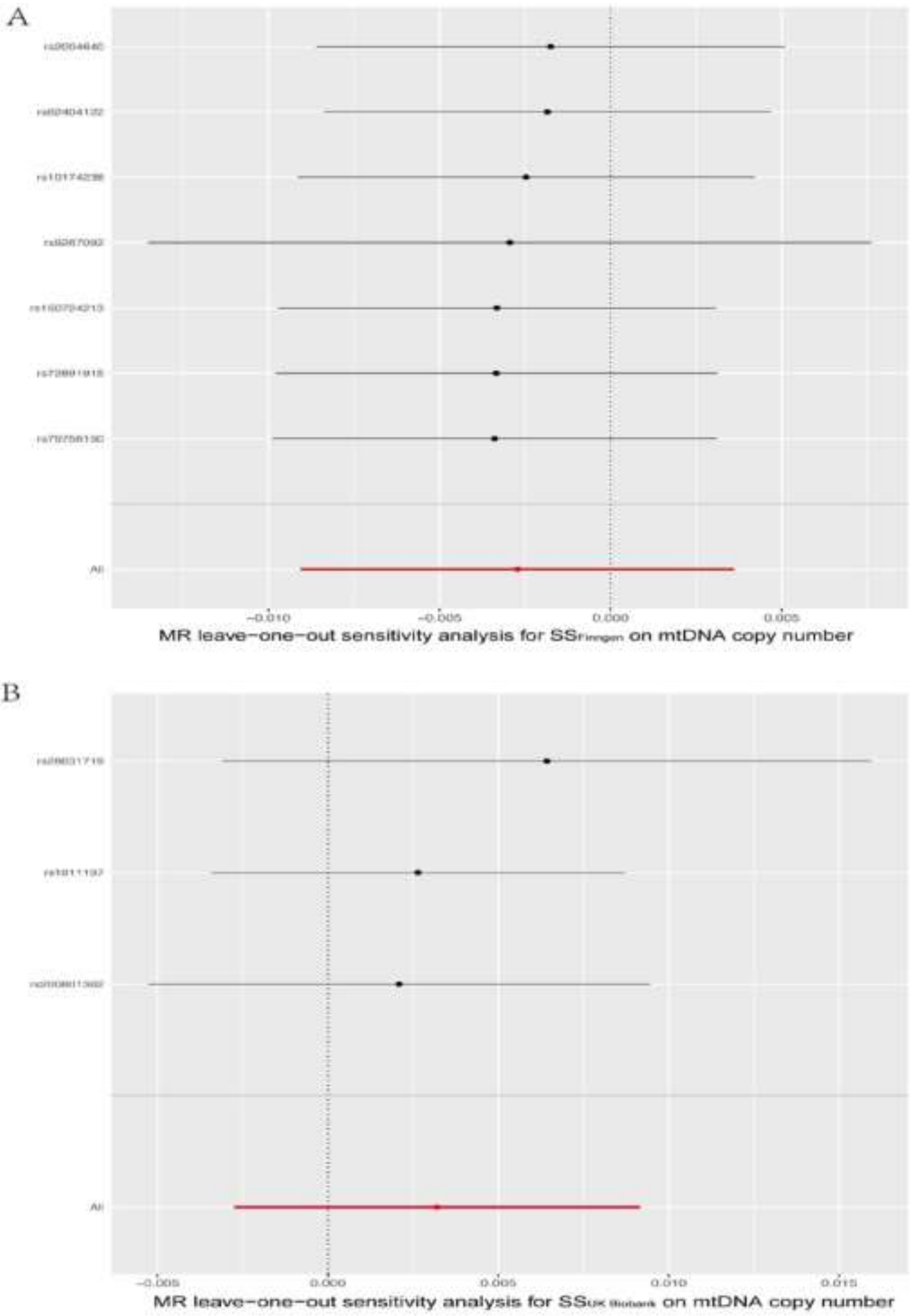

Supplementary Figure S4: Leave-one-out plot to visualize causal effect of SS risk on mtDNA copy number when leaving out one SNP in the validation cohort. A, SS data from FinnGen; B, SS data from UK Biobank; mtDNA, mitochondrial DNA; SS, Sjogren's syndrome; MR, Mendelian randomization.
